# Using the Electronic Medical Record to Reduce Unnecessary Ordering of Coagulation Studies for Patients with Chest Pain

**DOI:** 10.5811/westjem.2016.12.31927

**Published:** 2017-01-30

**Authors:** Jeremiah S. Hinson, Binoy Mistry, Yu-Hsiang Hsieh, Nicholas Risko, David Scordino, Karolina Paziana, Susan Peterson, Rodney Omron

**Affiliations:** Johns Hopkins University School of Medicine, Department of Emergency Medicine, Baltimore, Maryland

## Abstract

**Introduction:**

Our goal was to reduce ordering of coagulation studies in the emergency department (ED) that have no added value for patients presenting with chest pain. We hypothesized this could be achieved via implementation of a stopgap measure in the electronic medical record (EMR).

**Methods:**

We used a pre and post quasi-experimental study design to evaluate the impact of an EMR-based intervention on coagulation study ordering for patients with chest pain. A simple interactive prompt was incorporated into the EMR of our ED that required clinicians to indicate whether patients were on anticoagulation therapy prior to completion of orders for coagulation studies. Coagulation order frequency was measured via detailed review of randomly sampled encounters during two-month periods before and after intervention. We classified existing orders as clinically indicated or non-value added. Order frequencies were calculated as percentages, and we assessed differences between groups by chi-square analysis.

**Results:**

Pre-intervention, 73.8% (76/103) of patients with chest pain had coagulation studies ordered, of which 67.1% (51/76) were non-value added. Post-intervention, 38.5% (40/104) of patients with chest pain had coagulation studies ordered, of which 60% (24/40) were non-value added. There was an absolute reduction of 35.3% (95% confidence interval [CI]: 22.7%, 48.0%) in the total ordering of coagulation studies and 26.4% (95% CI: 13.8%, 39.0%) in non-value added order placement.

**Conclusion:**

Simple EMR-based interactive prompts can serve as effective deterrents to indiscriminate ordering of diagnostic studies.

## INTRODUCTION

Healthcare expenditures have risen sharply in the United States over the past decade and now account for one-fifth of the gross domestic product.^1^ With annual healthcare costs above $2.8 trillion and still rising, they represent a threat to national economic security and are a leading cause of individual financial hardship and bankruptcy. In light of this, recent estimates that up to 30% of healthcare expenditures are unnecessary and do not improve care are especially sobering.^1^,^2^ The need for increased value in U.S. healthcare is clear.

Physician decisions drive approximately 80% of healthcare expenditures, and many have suggested targeting clinician behaviors to reduce waste in U.S. healthcare.^1^,^2^ Multiple medical specialty societies have committed to this goal and as part of the Choosing Wisely Campaign have identified and targeted specific tests, treatments or services that are commonly used but are of little or no added value to the patient.^2^

More than five million patients undergo emergency department (ED) evaluation for chest pain in the U.S. annually.^3^ Once considered routine in the evaluation for chest pain, coagulation studies have been shown to lack utility in the absence of specific indications that include ongoing warfarin therapy, ST-elevation myocardial infarction, active bleeding, history of cirrhosis, and known or suspected coagulopathy.^4^,^5^ However, tests of prothrombin time (PT) and partial thromboplastin time (PTT) continue to be ordered frequently in the absence of these indications and account for more than $100 million in annual ED costs with no added value for the patient.^5^ Our goal was to reduce ordering of coagulation studies that have no added value for patients presenting with chest pain. We hypothesized this could be achieved via implementation of a stopgap measure in the electronic medical record (EMR) that gives providers deliberate feedback and allows for real-time reflection on the utility of ordering a test that may not be clinically indicated.

## METHODS

### Study Design and Setting

We conducted a pre and post quasi-experimental study to evaluate the impact of an EMR-based intervention on coagulation study ordering for patients with chest pain. The study was performed in the ED of a 1,059-bed tertiary care hospital with a comprehensive cardiovascular care center. This work was performed as a quality improvement initiative and was granted exempt status by our institutional review board.

### Description of Intervention

In August 2014, an electronic interactive prompt was incorporated into the EMR (EPIC) of our ED and set to appear each time a coagulation study (PT or PTT) was ordered. This prompt, which remained in place throughout the remainder of our study period, required ordering clinicians to indicate which anticoagulant therapy, if any, the patient was receiving prior to completion of the order using a series of two mouse clicks.

### Data Source and Sample Selection

Electronic records were retrieved for all ED patients with a chief complaint of chest pain during a two-month period before (May–June 2014) and after (October–November 2014) the intervention. We excluded a two-month washout period post-intervention to allow for normalization of the effect of the intervention. A systematic random sample of charts was generated for detailed review from each time period by selecting every seventh encounter. Reviewers annotated whether coagulation studies were ordered at time of initial ED evaluation and, if ordered, whether any clinical indication for the order existed. Clinical indications for coagulation study were defined as home vitamin K antagonist therapy, ST-elevation myocardial infarction, history of or suspicion for liver disease, known coagulopathy, initiation of anticoagulant therapy during ED treatment, or strong suspicion for vascular hemorrhage or stroke. We classified orders for patients not meeting these criteria as non-value added.

### Sample Size Determination and Statistical Analysis

We derived a sample size of at least 98 patients from each study period to detect an absolute 20% reduction in coagulation study order frequency from a baseline frequency of 75% with a confidence interval (CI) of 95% and power of 0.80. We calculated absolute difference and its corresponding 95% CI in the comparison of frequencies of total and non-value added coagulation study orders before and after intervention using chi-square test (SAS version 9.04, Cary, NC).

## RESULTS

There were 727 patient visits with a chief complaint of chest pain during the two-month pre-intervention sampling period and 822 during the post-intervention sampling period. We performed detailed chart review for a randomized selection of 103 visits pre-intervention and 104 visits post-intervention. Demographics were similar between groups with a mean age of 48 years in the pre-intervention group and 44 years in the post-intervention, and 53% male pre-intervention and 58% male post-intervention. Pre-intervention, 73.8% (76/103) of patients with chest pain had coagulation studies ordered, of which 67.1% (51/76) were non-value added with an overall rate of 49.5% (51/103) of patients having coagulation studies that added no value to their care. Post-intervention, only 38.5% (40/104) of patients with chest pain had coagulation studies ordered, of which 60% (24/40) were non-value added. Overall, only 23.1% (24/104) of patients had coagulation study orders that added no value to their care post-intervention. There was an absolute reduction of 35.3% (95% CI: 22.7%, 48.0%) in the total ordering of coagulation studies and 26.4% (95% CI: 13.8%, 39.0%) in non-value added order placement. The intervention increased the overall proportion of ordered tests that were value-added.

## DISCUSSION

Here, we show that a simple EMR-based intervention served as an effective deterrent to the ordering of non-value added diagnostic studies. While previous studies have shown that EMR-based interventions can lead to changes in clinician behavior, these interventions focused on more robust clinical decision support including display of evidence-based guidelines and individual diagnostic study costs.^6^–^8^ This intervention generated a short pause in clinician workflow, and required clinicians to reflect on the reasoning behind order placement. As a result, indiscriminate ordering was curtailed significantly.

This intervention led to significant estimated cost savings. Using standard Medicare reimbursement rates^9^ we estimated the average annual cost of coagulation studies on chest pain patients in our ED alone to be $47,959, of which $32,185 is non-value added. The intervention yielded a total annual cost savings of $22,964. Extrapolating these numbers to the national level demonstrates significant ongoing costs and potential for real savings. With over five million chest pain visits per year to EDs nationally,^3^ using the standard Medicare reimbursement rate to value these tests and assuming similar ordering behavior at other EDs, nearly $50 million is spent annually of which over $33 million is non-value added. Implementing a similar intervention nationwide could produce a cost savings of about $18 million on an annual basis.

## LIMITATIONS

While our findings strongly suggest that simple EMR-based interventions can alter clinician behavior and are potentially valuable tools for curtailing waste, there are important limitations to this work. We did not randomize patient encounters to EMR-based intervention, and comparisons were drawn between encounters that occurred before and after intervention. It is possible that other temporally related factors impacted clinician ordering patterns. Similarly, our ability to discern clinician motivation for decreased order frequency was limited to factors recorded in the EMR. For example, it is possible that the effect of our intervention was due to mouse-click fatigue, rather than improved decision-making. Indeed, we observed reductions in overall order frequency, and while our study was not designed to detect this, it is possible that this intervention resulted in decreased orders for coagulation studies that were clinically indicated. However, it is also possible that many of the tests we considered value-added did not provide any clinical contribution to care. For these reasons, EMR-based interventions such as this one are likely best paired with provider education initiatives. Finally, this work was performed at a single site and may not be directly applicable to all ED environments.

## CONCLUSION

Simple EMR-based interactive prompts can serve as effective deterrents to indiscriminate ordering of diagnostic studies.
